# G-T haplotype established by rs3785889-rs16941382 in *GOSR2* gene is associated with coronary artery disease in Chinese Han population

**DOI:** 10.18632/oncotarget.19280

**Published:** 2017-07-17

**Authors:** Shuo Pan, Gong-Chang Guan, Ying Lv, Zhong-Wei Liu, Fu-Qiang Liu, Yong Zhang, Shun-Ming Zhu, Rong-Huai Zhang, Na Zhao, Shuang Shi, Tomohiro Nakayama, Jun-Kui Wang

**Affiliations:** ^1^ First Department of Cardiology, People’s Hospital of Shaanxi Province, Xi’an, People’s Republic of China; ^2^ Division of Laboratory Medicine, Department of Pathology and Microbiology, Nihon University School of Medicine, Tokyo, Japan

**Keywords:** coronary artery disease, GOSR2, single nucleotide polymorphism, haplotype, genetic

## Abstract

**Objectives:**

The aim of the present study is to assess the association between the human *GOSR2* gene and coronary artery disease using a haplotype-based case-control study in Chinese Han population.

**Methods:**

A total of 283 coronary artery disease patients and 280 controls were genotyped for the human *GOSR2* gene (rs197932, rs3785889, rs197922, rs17608766, and rs16941382). Data were analyzed for three separate groups: the total subjects, men, and women.

**Results:**

For the total subjects, the frequency of the G-T haplotype established by rs3785889-rs16941382 was significantly higher in the coronary artery disease patients as compared to the control subjects (*P*=0.009). Multiple logistic regression analysis also confirmed that the subjects with G-T haplotype established by rs3785889-rs16941382 (homozygote) were found having significantly higher chance suffering from coronary artery disease than the ones without this haplotype (OR=1.887, *P*=0.007).

**Conclusions:**

The G-T haplotype established by rs3785889-rs16941382 may be a risk genetic marker for coronary artery disease patients in Chinese Han population.

## INTRODUCTION

Coronary artery disease (CAD) is a widely spread disease worldwide, its pathogenesis includes both environmental and genetic factors [1], many hereditary features were reported to show significant connections with CAD in previous investigations [2–3].

Golgi Snap receptor complex member 2 (GOSR2) is one of the Golgi-associated soluble N-ethylmaleimide-sensitive factor attachment receptor (SNARE) proteins, its main function included the transportation via Golgi complex in different cells [4]. The function also includes interaction with target-localized SNAREs (t-SNAREs), with which the insulin, leptin, angiotensinogen and other macromolecules would go through Golgi compartments [5–7].

One study based on American population reported that the A allele in rs197922 of *GOSR2* gene was a risk factor for myocardial infarction (OR=1.17, *P*=0.032) [8]. Pan et al [9] did not find A allele was an significant risk or protective factor for MI, however, base on Japanese population, the T-G-G haplotype of rs197932-rs3785889-rs197922 in *GOSR2* was found to show beneficial effects against the occurrences of myocardial infarction.

However, in Chinese Han population, there is no such study to evaluate the relationship between *GOSR2* gene and CAD. Therefore, the present paper is intended to discover the possible connection between the human *GOSR2* gene and CAD in Chinese Han population.

## RESULTS

Table 1 presented the dimorphic and laboratory data in patients with or without CAD. For total subjects, fasting glucose and incidence of diabetes in CAD patients were higher than those in the control subjects significantly (*P*<0.05). For men, body mass index, high density lipoprotein (HDL) and fasting glucose in CAD patients were different with those in control subjects significantly (*P*<0.05). In women, body mass index, triglycerides, HDL, fasting glucose, creatinine and incidence of diabetes in CAD patients were different with those in control subjects significantly (*P*<0.05).

**Table 1:** Characteristics of study participants

	Total			Men			Women		
	CAD patients	Control subjects	*P* value	CAD patients	Control subjects	*P* value	CAD patients	Control subjects	*P* value
Number of subjects	283	280		134	151		149	129	
Age (years)	56.56±11.28	56.60±12.52	0.907	55.51±8.75	55.60±12.41	0.885	58.48±14.80	58.41±12.55	0.834
BMI (kg/m^2^)	25.45±3.08	25.89±3.17	0.119	23.12±1.76	26.23±2.91	<0.001*	27.90±2.12	25.49±3.42	<0.001*
Pulse (beats/min)	72.90±11.30	73.34±10.11	0.632	73.08±12.27	74.55±10.92	0.289	72.74±10.39	71.91±9.89	0.480
Total cholesterol (mmol/L)	4.28±1.04	4.41±1.03	0.132	4.25±1.12	4.31±1.03	0.637	4.30±0.97	4.52±1.02	0.067
Triglycerides (mmol/L)	2.04±1.78	1.94±1.33	0.430	1.93±2.05	2.17±1.57	0.285	2.14±1.51	1.67±0.92	0.003*
LDL (mmol/L)	2.56±0.93	2.56±8.77	0.972	2.47±0.94	2.52±0.85	0.642	2.65±0.92	2.61±0.91	0.719
HDL (mmol/L)	1.16±0.34	1.15±0.39	0.749	1.25±0.40	1.08±0.41	0.001*	1.08±0.24	1.23±0.35	<0.001*
Fasting glucose (mmol/L)	6.21±2.39	5.67±1.83	0.003*	6.36±2.47	5.75±2.04	0.026*	6.08±2.31	5.57±1.56	0.033*
Creatinine (mmol/L)	74.88±24.07	71.65±17.60	0.058	74.61±21.34	76.41±17.17	0.092	75.13±22.77	66.15±16.51	<0.001*
Hypertension (%)	183(64.66%)	168(60.00%)	0.259	89(66.42%)	95(62.91%)	0.620	94(63.09%)	73(56.59%)	0.326
DM (%)	107(37.81%)	50(17.86%)	<0.001*	42(31.34%)	33(21.85%)	0.080	65(43.62%)	17(13.18%)	<0.001*

Continuous variables are expressed as mean ± S.D. Categorical variables are expressed as percentages. CAD, coronary artery disease; BMI, body mass index; LDL, low-density lipoprotein; HDL, high-density lipoprotein; DM, diabetes mellitus. The P value of the continuous variables was calculated by the Mann-Whitney U-test. The P value of the categorical variables was calculated by Fisher’s exact test. *P < 0.05.

Table 2 presents the genotyping and allele frequency as well as the frequencies in dominant and recessive models between the CAD patients and control subjects for each SNP. For each single nucleotide polymorphism (SNP), the frequency of genotype was consistent with Hardy-Weinberg equilibrium (data not shown). The genotype distributions showed no significant difference for the all 5 SNPs in *GOSR2*. For total, men and women subjects, the distribution frequency showed no significant difference in the allele distribution as well as the dominant and recessive model distribution.

**Table 2:** Genotyping and allele distributions in control subjects and patients with CAD

Variants	Total			Men			Women		
	CAD patients	Control subjects	*P* value	CAD patients	Control subjects	*P* value	CAD patients	Control subjects	*P* value
rs197932(SNP1)									
Genotyping									
CC	27(9.5%)	17(6.1%)		14(10.4%)	6(4.0%)		13(8.7%)	11(8.5%)	
CT	112(39.6%)	115(41.1%)		51(38.1%)	63(41.7%)		61(40.9%)	52(40.3%)	
TT	144(50.9%)	148(52.9%)	0.324	69(51.5)	82(54.3%)	0.109	75(50.3%)	66(51.2%)	1.000
Dominant model									
TT	144(50.9%)	148(52.9%)		69(51.5%)	82(54.3%)		75(50.3%)	66(51.2%)	
CC+CT	139(49.1%)	132(47.1%)	0.673	65(48.5%)	69(45.7%)	0.721	74(49.7%)	63(48.8%)	0.905
Recessive model									
CC	27(9.5%)	17(6.1%)		14(10.4%)	6(4.0%)		13(8.7%)	11(8.5%)	
TT+CT	256(90.5%)	263(93.9%)	0.157	120(89.6%)	145(96.0%)	0.068	136(91.3%)	118(91.5%)	1.000
Allele									
C	166(29.3%)	149(26.6%)		79(29.5%)	75(24.8%)		87(29.2%)	74(28.7%)	
T	400(70.7%)	411(73.4%)	0.320	189(70.5%)	227(75.2%)	0.221	211(70.8%)	184(71.3%)	0.925
rs3785889(SNP2)									
Genotyping									
AA	27(9.5%)	26(9.3%)		11(8.2%)	15(9.9%)		16(10.7%)	11(8.5%)	
AG	105(37.1%)	127(45.4%)		50(37.3%)	68(45.0%)		55(36.9%)	59(45.7%)	
GG	151(53.4%)	127(45.4%)	0.124	73(54.5%)	68(45.0%)	0.286	78(52.3%)	59(45.7%)	0.320
Dominant model									
GG	151(53.4%)	127(45.4%)		73(54.5%)	68(45.0%)		78(52.3%)	59(45.7%)	
AA+AG	132(46.6%)	153(54.6%)	0.064	61(45.5%)	83(55.0%)	0.124	71(47.7%)	70(54.3%)	0.282
Recessive model									
AA	27(9.5%)	26(9.3%)		11(8.2%)	15(9.9%)		16(10.7%)	11(8.5%)	
GG+AG	256(90.5%)	254(90.7%)	1.000	123(91.8%)	136(90.1%)	0.683	133(89.3%)	118(91.5%)	0.551
Allele									
A	159(28.1%)	179(32.0%)		72(26.9%)	98(32.5%)		87(29.2%)	81(31.4%)	
G	407(71.9%)	381(68.0%)	0.172	196(73.1%)	204(67.5%)	0.169	211(70.8%)	177(68.6%)	0.580
rs197922(SNP3)									
Genotyping									
AA	43(15.2%)	38(13.6%)		27(20.1%)	24(15.9%)		16(10.7%)	14(10.9%)	
AG	125(44.2%)	139(49.6%)		57(42.5%)	71(47.0%)		68(45.6%)	68(52.7%)	
GG	115(40.6%)	103(36.8%)	0.436	50(37.3%)	56(37.1%)	0.590	65(43.6%)	47(36.4%)	0.437
Dominant model									
AG	125(44.2%)	139(49.6%)		57(42.5%)	71(47.0%)		68(45.6%)	68(52.7%)	
AA+GG	158(55.8%)	141(50.4%)	0.206	77(57.5%)	80(53.0%)	0.475	81(54.4%)	61(47.3%)	0.279
Recessive model									
AA	43(15.2%)	38(13.6%)		27(20.1%)	24(15.9%)		16(10.7%)	14(10.9%)	
GG+AG	240(84.8%)	242(86.4%)	0.632	107(79.9%)	127(84.1%)	0.358	133(89.3%)	115(89.1%)	1.000
Allele									
A	211(37.3%)	215(38.4%)		111(41.4%)	119(39.4%)		100(33.6%)	96(37.2%)	
G	355(62.7%)	345(61.6%)	0.713	157(58.6%)	183(60.6%)	0.669	198(66.4%)	162(62.8%)	0.375
rs17608766(SNP4)									
Genotyping									
TT	281(99.3%)	277(98.9%)		134(100%)	149(98.7%)		147(98.7%)	128(99.2%)	
CT	2(0.7%)	3(1.1%)		0(0%)	2(1.3%)		2(1.3%)	1(0.8%)	
CC	0(0%)	0(0%)	0.685	0(0%)	0(0%)	0.500	0(0%)	0(0%)	1.000
Dominant model									
TT	281(99.3%)	277(98.9%)		134(100%)	149(98.7%)		147(98.7%)	128(99.2%)	
CC+CT	2(0.7%)	3(1.1%)	0.685	0(0%)	2(1.3%)	0.500	2(1.3%)	1(0.8%)	1.000
Recessive model									
CC	0(0%)	0(0%)		0(0%)	0(0%)		0(0%)	0(0%)	
TT+CT	283(100%)	280(100%)	-	134(100%)	151(100%)	-	149(100%)	129(100%)	-
Allele									
C	2(0.4%)	3(0.5%)		0(0%)	2(0.7%)		2(0.7%)	1(0.4%)	
T	564(99.6%)	557(99.5%)	0.685	268(100%)	300(99.3%)	0.501	296(99.3%)	257(99.6)	1.000
rs16941382(SNP5)									
Genotyping									
CC	21(7.4%)	22(7.9%)		15(11.2%)	17(11.3%)		6(4.0%)	5(3.9%)	
CT	100(35.3%)	121(43.2%)		43(32.1%)	63(41.7%)		57(38.3%)	58(45.0%)	
TT	162(57.2%)	137(48.9%)	0.132	76(56.7%)	71(47.0%)	0.218	86(57.7%)	66(51.1%)	0.564
Dominant model									
TT	162(57.2%)	137(48.9%)		76(56.7%)	71(47.0%)		86(57.7%)	66(51.2%)	
CC+CT	121(42.8%)	143(51.1%)	0.052	58(43.3%)	80(53.0%)	0.123	63(42.3%)	63(48.8%)	0.280
Recessive model									
CC	21(7.4%)	22(7.9%)		15(11.2%)	17(11.3%)		6(4.0%)	5(3.9%)	
TT+CT	262(92.6%)	258(92.1%)	0.875	119(88.8%)	134(88.7%)	1.000	143(96.0%)	124(96.1%)	1.000
Allele									
C	142(25.1%)	165(29.5%)		73(27.2%)	97(32.1%)		69(23.2%)	68(26.4%)	
T	424(74.9%)	395(70.5%)	0.108	195(72.8%)	205(67.9%)	0.233	229(76.8%)	190(73.6%)	0.430

CAD, coronary artery disease; SNP, single-nucleotide polymorphism. *P < 0.05.

The linkage disequilibrium data of the selected 4 SNPs are presented in Table 3. All the 5 SNPs were confirmed to be placed in the same haplotype block according to the | D'| values. As the frequency of C allele in SNP4 was too low for the haplotype construction. Meanwhile, since *r*^2^ for SNP3-SNP5 was 0.672, which was above 0.5 in the participants, indicating that SNP3 and SNP5 may not be applied for constructing haplotypes at the same time. Considering that the genotype distribution difference in SNP5 was more significant than that in SNP3, we chose SNP1, SNP2, and SNP5 to construct the haplotype.

**Table 3:** Pairwise linkage disequilibrium for four SNPs

	| D’| values				
		SNP1	SNP2	SNP3	SNP5
**r**^2^ **values**	SNP1		0.248	0.836	0.958
	SNP2	0.010		1.000	0.968
	SNP3	0.158	0.293		0.967
	SNP5	0.139	0.184	0.627	

SNP, single nucleotide polymorphism. | D'| values of >0.5 to assign SNP locations to one haplotype block. SNPs with an *r*^2^ value > 0.5 were selected as tagged. | D'| above the diagonal and r^2^ below the diagonal. The shadowed portion indicates | D'| > 0.5 and r^2^ >0.5.

Table 4 presented thehaplotype-based analysis via the establishment of haplotype using SNP1-SNP2-SNP5, SNP1-SNP2, SNP1-SNP5, SNP2-SNP5. In total subjects, the overall SNP2-SNP5 haplotype distribution differed significantly between the CAD patients and the controls (*P*=0.031). In total subjects, the G-T haplotype frequency constructed via SNP2-SNP5 in the CAD patients was higher than that in the controls (0.468 vs 0.389, *P* = 0.009). Meanwhile, in men, the G-T haplotype frequency constructed via SNP2-SNP5 in CAD patients was also higher than that in control subjects (0.459 vs 0.360, *P* = 0.021). As in women, the same distribution patterns were not noticed.

**Table 4:** Haplotype analysis in patients with CAD and control subjects

Haplotype	SNP1	SNP2	SNP5	Overall *P* value			Frequency in total			Frequency in men			Frequency in women		
				Total	Men	Women	CAD patients	Control subjects	*P* value	CAD patients	Control subjects	*P* value	CAD patients	Control subjects	*P* value
				0.149	0.278	0.445									
H1	T	G	C				0.248	0.291	0.092	0.272	0.315	0.273	0.226	0.264	0.265
H2	T	A	T				0.229	0.251	0.435	0.207	0.261	0.138	0.248	0.238	0.804
H3	C	G	T				0.239	0.198	0.126	0.233	0.188	0.243	0.245	0.211	0.354
H4	T	G	T				0.231	0.192	0.147	0.226	0.175	0.146	0.235	0.212	0.552
H5	C	A	T				0.053	0.068	0.342	0.062	0.061	0.962	0.046	0.076	0.153
				0.425	0.599	0.589									
H6	T	G					0.476	0.478	0.930	0.490	0.481	0.864	0.463	0.474	0.798
H7	T	A					0.231	0.256	0.416	0.215	0.271	0.195	0.245	0.239	0.897
H8	C	G					0.244	0.202	0.137	0.241	0.195	0.301	0.245	0.212	0.382
H9	C	A					0.050	0.064	0.447	0.054	0.054	0.989	0.047	0.075	0.288
				0.238	0.351	0.557									
H10	T		T				0.459	0.443	0.579	0.433	0.437	0.908	0.483	o.450	0.461
H11	C		T				0.293	0.265	0.304	0.295	0.247	0.197	0.291	0.287	0.902
H12	T		C				0.248	0.292	0.094	0.272	0.317	0.279	0.226	0.264	0.251
				0.031*	0.067	0.400									
H13		G	T				0.468	0.389	0.009*	0.459	0.360	0.021*	0.477	0.423	0.181
H14		A	T				0.281	0.317	0.210	0.269	0.320	0.180	0.292	0.314	0.511
H15		G	C				0.251	0.294	0.101	0.272	0.320	0.238	0.232	0.264	0.348

CAD, coronary artery disease; SNP, single-nucleotide polymorphism. Haplotype with frequencies >0.01 were estimated using SNPAlyze software. *P* value was calculated by permutation test using the bootstrap method. *P < 0.05.

The software SNPAlyze version 8.2 was applied to construct the diplotypes for every single patient. The H13 (homozygote) was taken into the logistic regression analysis as one potential risk factor, other confounding such as diabetes mellitus, hypertension, low density lipoprotein (LDL), total cholesterol, triglycerides and creatinine were also taken into the logistic regression analysis as adjusting factors (Table 5). In total subjects, after the adjustments of hypertension, diabetes mellitus, low density lipoprotein (LDL), total cholesterol, triglycerides and creatinine, the subjects carrying the H13 diplotype (H13 homozygote) were shown to have increased probability of suffering from CAD than the those not carrying H13 diplotype (homozygote) (*OR*=1.887, *P*=0.007).

**Table 5:** Odds ratios and 95% confidence intervals (CI) for each confounding factor and haplotype associated with CAD

Risk factors	Odd ratios	95%CI	*P* value
H13 haplotype (homozygote)	1.887	1.188-2.995	0.007*
DM	2.606	1.731-3.922	<0.001*
Hypertension	1.093	0.758-1.576	0.634
LDL	1.385	1.013-1.894	0.041*
TC	1.542	1.134-2.077	0.026*
TG	1.121	0.980-1.283	0.096
Creatinine	1.006	0.997-1.016	0.178

CAD, coronary artery disease; DM, diabetes mellitus; LDL, low-density lipoprotein; TC, total cholesterol; TG, triglycerides; *P < 0.05.

## DISCUSSION

GOSR2 is a Golgi associated soluble factor attachment receptor (SNARE) protein, it built bridges to allow and promote membrane fusion via the endosomal and secretory pathways [10, 11]. A twisted parallel bundle constructed by four amphipathic helices were contained in the SNARE complex [12, 13]. Although the specific function of SNAREs, such as transport vesicles functions of docking and fusion, is still uncertain, some studies confirmed that SNARE complexes played the key part in intracellular membrane fusion. The trafficking function of cardiovascular related macromolecules may be in the central stage for the association with multiple cardiovascular diseases such as CAD and hypertension [9, 14].

In one previous study based on the Japanese population, TT genotype is the only genotype in the rs17608766 of *GOSR2* [9]. In this study based on Chinese Han population, we found 2 subjects had the CT genotype in the CAD group and 3 subjects had the CT genotype in the control group in Chinese Han population. However, we did not notice the significant difference on CT and TT genotype distribution between the CHD patients and control subjects. One study based on American population reported that the A allele in rs197922 of *GOSR2* gene was a risk factor for myocardial infarction (MI) (OR=1.17, *P*=0.032) [8]. However, the study base on the Japanese population reported no significant difference for A allele in rs197922 between the control subjects and CAD patients. In this study based on Chinese Han population, the frequency of A allele in rs197922 of *GOSR2* gene didn’t show any significant difference between the control subjects and CAD patients (*P*=0.713). Our results were consistent with the findings in Japanese population and were not consistent with the findings in American population. The differences may mainly be due to the races, since the Japanese population was more likely to be consistent in the origin with Chinese populations.

When the linkage disequilibrium among the SNPs appeared to be weak, the haplotypes analysis could be superior than individual SNPs analysis since it is consist with the human hereditary features [15]. In the previously described study based on Japanese population, after constructing all the possible haplotypes, they discovered that the frequency distribution of T-G-G haplotype, which was constructed with rs197932-rs3785889-rs197922, was lower in the myocardial infarction men when compared with that in the control men (*P*=0.040). After adjusting the confounding factors, they found that the T-G-G haplotype including homozygous and heterozygous diplotypes may have protective effects on myocardial infarction in Japanese men (OR=0.455, *P*=0.041) [9]. In this present study, we did not use the rs197932-rs3785889-rs197922 to establish the halotype since the genotype distribution difference in rs16941382 was more significant than that in rs197922 in Chinese population. Therefore, we constructed the haplotype using rs197932-rs3785889-rs16941382 and found a risk haplotype G-T of SNP2-SNP5 in total and male population. With the logistic regression analysis, the haplotype (G-T) is targeted as an novel risk factor for CAD in Chinese Han population (*OR*=1.887, *P*=0.007).

The mechanism that *GOSR2* is associated with CAD is still not clear. Based on limited data, we supposed that three possible mechanisms may be involved in the association between the *GOSR2* and CAD. First, the genome-wide association study (GWAS) showed that rs17608766 in *GOSR2* had age-dependent effects on BP [16]. The association with blood pressure may accelerate the arteriosclerosis which would consequently make the some haplotypes in *GOSR2* gene the risk factor of CAD. Second, Ghanbari M et al [17] identified that *GOSR2* was one of the miR-4513 target genes, which may show multiple effects on lipid and glucose metabolism, blood pressure regulation as well as atherosclerosis. Then the association between *GOSR2* and coronary artery disease may be established via miRNA-4513 pathway. Third, GOSR2 has the function of coding for vesicular-SNARE (v-SNAREs), which was perceived as transporter of vesicles into the Golgi complex [18]. v-SNAREs would have influence on target-localized SNAREs (t-SNAREs), allowing the macromolecule substances to move directly across Golgi membrane [19, 20]. Due to its trafficking function, the *GOSR2* gene could be associated with the cardiovascular diseases which are highly associated with macromolecules such as insulin, leptin, and angiotensinogen.

Our study has several strengths. First, all case and control subjects underwent the coronary angiography, which is the golden standard for the diagnosis of CAD with the combination of clinical or electrocardiogram results. Second, the control subjects may have other cardiovascular or metabolic or endocrine diseases, for example, they may have hypertension, diabetes mellitus (DM), and lipids disorders, which meant that the subjects in control group may share the same risk factors as subjects in CAD group except for that the results of coronary angiography were normal. It could decrease the effects of environmental factors of CAD, increase the homogeneity of the two groups and focus mainly on the effects of genetic factors.

There are some limitations in the present study. In Chinese women of this study, the G-T haplotype frequency constructed by SNP2-SNP5 was higher in CAD patients when compared with controls, but the *P* value did not reach significance (*P*=0.181). We believed the enlargement in women samples may lead to more significant difference.

At last, the association between the human *GOSR2* gene and CAD has been addressed for the first time in the Chinese Han population. The data indicates that in Chinese Han population, the G-T haplotype established by rs3785889-rs16941382 of the human *GOSR2* gene might be the risk marker for coronary artery disease.

## MATERIALS AND METHODS

### Ethics statement

Written informed consent was obtained from all participants. All participants explicitly provided permission for DNA analyses as well as collection of relevant clinical data. This study was approved by the Ethics Committee of People's Hospital of Shaanxi Province. It was conducted according to the standards of the Declaration of Helsinki. Authors have access to information that could identify individual participants during or after data collection.

### Subjects

The subjects were from the Han population who lived in Shaanxi. All patients and controls had a differential diagnosis for chest pain encountered in the Cardiac Catheterization Department of People's Hospital of Shaanxi Province from January in 2015 to December in 2016. The procedures of coronary angiography were undertaken by highly skilled physicians using the Judkins approach. The findings of coronary angiography were interpreted by at least two experienced imaging specialists and the final diagnosis of CAD was made according to the angiography report.

We randomly recruited 283 Han patients with CAD and 280 age matched controls. The CAD was defined as the presence of at least one significant coronary artery stenosis of more than 50% luminal diameter in coronary angiography. All control subjects also underwent the coronary angiography and had no coronary artery stenosis and did not show clinical or electrocardiogram evidence of myocardial infarction or CAD. Control subjects were not healthy individuals, some of them had hypertension, some of them had diabetes mellitus (DM), and some of them had lipids disorders, which meant that the subjects in control group were exposed to the same risk factors as subjects in CAD group except that the results of coronary angiography were normal.

The data of traditional coronary risk factors, including blood lipids, hypertension and DM were collected from all study participants. The diagnosis of hypertension was established if patients were on antihypertensive medication or if the mean of 3 measurements of systolic blood pressure (SBP) ≥140 mm Hg or diastolic blood pressure (DBP) ≥90 mm Hg, respectively. Diabetes mellitus was defined on the basis of the World Health Organization (WHO) criteria. In addition, individuals with fasting plasma glucose ≥7.0 mmol/L or with a history of diabetes or treatment with insulin were considered diabetic. All patients with impaired renal function, malignancy, connective tissue disease, valvular disease or chronic inflammatory disease were excluded.

### SNP selection

*GOSR2* gene in human has three transcript variants to encode three different isoforms (isoform A, isoform B, isoform C). The isoform A of *GOSR2* gene has the longest length among the three isoforms, it consists of 212 amino acids and is located on chromosome 17q21. This gene is approximately 18.25 kilobase pairs (kbp) and contains six exons separated by five introns.

In this study, we screened the data on the International HapMap Project website (http://hapmap.ncbi.nlm.nih.gov/index.html.en) for the Tag SNPs of *GOSR2* gene. SNPs with relatively high minor allele frequencies (MAF) have been shown to be useful as genetic markers in genetic association studies, so we selected three SNPs (rs3785889, rs197932, and rs16941382) which had a MAF of >0.1 as the tag SNPs in Chinese population. rs197932 is located 26 kbp upstream from the start codon in exon 1, while rs16941382 is located 25 kbp downstream from the stop codon in exon six. Meanwhile, we also include rs17608766 and rs197922 which may be associated with blood pressure and coronary heart disease respectively in *GOSR2* gene [8, 21].

We designated the five SNPs as SNP1 (rs197932, C_2592633_10), SNP2 (rs3785889, C_2960489_10), SNP3 (rs197922, C_2275273_10), SNP4 (rs17608766, C_33589426_10), and SNP5 (rs16941382, C_33589395_10), which were in order of increasing distance from the 5′ end of the gene.

### Genotyping

Venous blood samples were collected from all participants in fasting state, and genomic DNA was extracted from the peripheral blood leukocytes using phenol and chloroform extraction method in Central Laboratory of People's Hospital of Shaanxi Province from January in 2015 to December in 2016 and stored in -80°C refrigerator for further genotyping [22, 23].

The DNA samples were carried on dry ice to Laboratory Department of Shanghai GENESKY Biological Technology Co., Ltd. (http://www.geneskies.com/) via airplane for genotyping in January in 2017. Genotyping was performed using the TaqMan SNP Genotyping Assay (Applied Biosystems). The primers and probes used in the TaqMan SNP Genotyping Assays (Applied Biosystems) were chosen based on information available at the ABI website (http://www.appliedbiosystems.com/AB_Home/index.htm).

Polymerase chain reaction (PCR) amplification was performed using 2.5 μl of TaqMan Universal Master Mix, No AmpErase UNG (2×) (Applied Biosystems) in a 5 μl final reaction volume, along with 2 ng DNA, 2.375 μl ultrapure water, 0.079 μl Tris-EDTA (TE) buffer (1×), 0.046 μl TaqMan SNP Genotyping Assay Mix (40×) containing a 331.2 nmol/l final concentration of primers and a 73.6 nmol/l final concentration of the probes. The thermal cycling conditions were as follows: 50°C for 2 min; 95°C for 10 min; 50 cycles of 95°C for 15 s; and 60°C for 1 min [24, 25]. The plates were read on the sequence detection system 7900 instrument with the end-point analysis mode of the sequence detection system version 1.6.3 software package (Applied Biosystems). The genotypes were determined visually based on the dyecomponent fluorescent emission data depicted in the X-Y scatter-plot of the sequence detection system software. The genotypes results were saved in two separate output files for later comparison [26].

### Statistical analysis

All continuous variables were expressed as mean ± S.D. Differences in continuous variables between the CAD patients and control subjects were analyzed using the Mann-Whitney *U*-test. Differences in categorical variables were analyzed using Fisher’s exact test. Differences in distributions of genotypes and alleles between CAD patients and control subjects were analyzed using Fisher’s exact test. Based on the genotype data of the genetic variations, we performed linkage disequilibrium and haplotype-based case-control analyses using the software SNPAlyze version 8.2 (Dynacom, Yokohama, Japan) [27]. The pairwise linkage disequilibrium analysis was performed using four SNP pairs. We used | D'| values of >0.5 to assign SNP locations to one haplotype block. SNPs with an *r*^2^ value > 0.5 were selected as tagged. In the haplotype-based case-control analysis, haplotypes with a frequency of <0.01 were excluded. Logistic regression analysis was performed to assess the contribution of the major risk factors after constructing diplotypes for each subject by SNPAlyze version 8.2 (Dynacom, Yokohama, Japan). Statistical significance was established at *P* < 0.05. Statistical analyses were performed using SPSS software for Windows, version 17.0 (SPSS, Chicago, IL).
